# Design and Synthesis of Hyperbranched Aromatic Polymers for Catalysis

**DOI:** 10.3390/polym10121344

**Published:** 2018-12-05

**Authors:** Yuta Nabae, Masa-aki Kakimoto

**Affiliations:** Department of Materials Science and Engineering, Tokyo Institute of Technology, 2-12-1 S8-26 Ookayama, Meguro-ku, Tokyo 152-8552, Japan; nabae.y.aa@m.titech.ac.jp

**Keywords:** hyperbranched polymer, aromatic polymer, solid acid catalyst, partial oxidation, AB_2_ monomer, A_2_ + B_3_ polymerization, heterogeneous catalyst, polycondensation

## Abstract

Aromatic polymers such as poly(ether sulfone), poly(ether ketone), and polyimide have been widely used in industry due to their thermal, mechanical, and chemical stabilities. Although their application to catalysis has been limited, the introduction of a hyperbranched architecture to such aromatic polymers is effective in developing catalytic materials that combine the advantages of homogenous and heterogeneous catalysts. This review article overviews the recent progress on the design and synthesis of hyperbranched aromatic polymers. Several acid catalyzed reactions and the aerobic oxidation of alcohols have been demonstrated using hyperbranched aromatic polymers as catalysts. The advantage of hyperbranched polymers against linear polymers is also discussed.

## 1. Introduction

Aromatic polymers such as poly(ether sulfone), poly(ether ketone), and polyimide are well-known for their thermal, chemical, and mechanical stabilities. Although they are widely used as high performance polymers in industry, their application to catalysis has been quite limited, most likely because such aromatic polymers are considered inert and hence unsuitable for catalysis. The development of catalytically active aromatic polymers will largely expand the applicability of organic and polymeric materials in catalysis.

**Figure 1:**
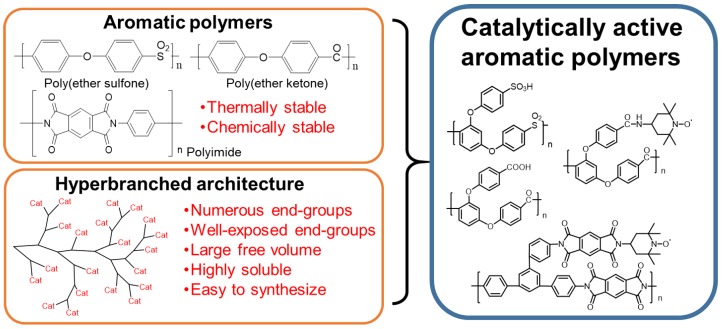
Concept of hyperbranched aromatic polymers for use in catalysis.

To develop catalytically active aromatic polymers, a hyperbranched architecture is of interest. Hyperbranched polymers have a dendritic structure with a large number of end-groups [1,2,3]. The synthesis of a hyperbranched polymer is easier than that of dendrimers as hyperbranched polymers can be synthesized in a one-step process from AB*_x_*-type monomers [4] where *x* is two or more. They can be also synthesized via A_2_ + B_3_ polymerizations [5]. As illustrated in Figure 1, hyperbranched architecture is suitable to develop catalytically active aromatic polymers, since a large number of catalytically active sites can be introduced by converting the end-groups into catalytically active functional groups. In addition, there are several reasons, given their less-entangled main chains, to assume that hyperbranched polymers would be suitable for use in catalysis as their catalytically active end-groups would be well-exposed and accessible to the reactants of catalytic reactions. A large free volume of hyperbranched polymers would contribute to the mass transport of the reactants and products. Additionally, hyperbranched polymers are well-soluble in many solvents. Typically, soluble homogenous catalysts exhibit a higher catalytic activity than that of heterogeneous catalysts, whereas the separation of the catalysts from the products is easier with heterogeneous catalysts. The good solubility of hyperbranched polymers would guarantee good catalytic activity, moreover, they can be used as heterogeneous catalysts after immobilization onto insoluble supports such as carbon materials. In other words, hyperbranched polymers can combine the advantages of homogenous and heterogeneous catalysts of high catalytic activity and ease of separation.

This review article focused on the recent progress in the development of hyperbranched aromatic polymers for use in catalysis. Poly(ether sulfone), poly(ether ketone), and polyimides with hyperbranched architectures have been synthesized, and some of them have been demonstrated as catalyst materials for various reactions such as esterification, hydrolysis of cellulose, and partial oxidation of alcohols.

## 2. Hyperbranched Poly(Ether Sulfone) for Catalysis

Many chemical reactions are catalyzed in acid; therefore, acid-catalyzed chemical reactions are very important in producing various chemicals. With regard to green chemistry, it is quite important to replace liquid acid catalysts such as HF and H_2_SO_4_ with solid acid catalysts [6]. Ion-exchanging polymers such as Amberlyst^®^ are typical solid acid catalysts; however, they are usable only at ambient operating temperatures typically below 120 °C because of the low thermal stability of the aliphatic main chains [7]. If ion-exchanging polymers with a higher thermal stability could be developed, the applicability of polymer-based solid acid catalysts would be largely expanded; aromatic hyperbranched poly(ether sulfone) could be a promising material.

The use of aromatic hyperbranched polymers in catalysis began from a sulfonated aromatic polymer. Van de Vyver et al. prepared sulfonated hyperbranched poly(arylene oxindole)s via the A_2_ + B_3_ polycondensation of isatin and 1,3,5-tri-(4-phenoxybenzoyl)-benzene, and demonstrated the hydrolysis of cellulose in a homogenous catalytic reaction [8]. More recently, as illustrated in Figure 1 and mentioned below [9,10], our research group demonstrated the use of hyperbranched poly(ether sulfone) as an acid catalyst.

### 2.1. Synthesis of HBSPES and HBSPES/CB

**Scheme 1:**
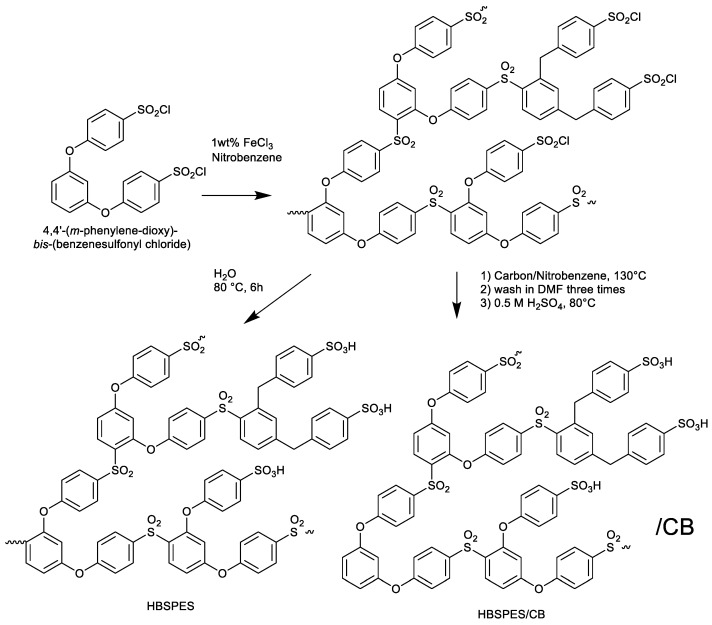
Synthesis route of HBSPES and HBSPES/CB [10].

Hyperbranched poly(ether sulfone) was synthesized from an AB_2_ monomer, 4,4’-(*m*-phenylene-dioxy)-*bis*-(benzenesulfonyl chloride), as illustrated in Scheme 1. This synthesis approach was designed for the development of a proton-exchange membrane [11,12,13], and our research group applied it to acid-catalyzed reactions [9,10]. Hyperbranched sulfonated poly(ether sulfone) (HBSPES) is itself soluble in many solvents, and therefore, will work as a homogenous catalyst. Conventional linear sulfonated poly(ether sulfone) (LSPES) was also prepared to compare the nature of the polymers as acid catalyst materials. The molecular weights (*M*_w_) of these polymers were adjusted in the range of 13–17 kDa by changing the polymerization conditions.

**Figure 2:**
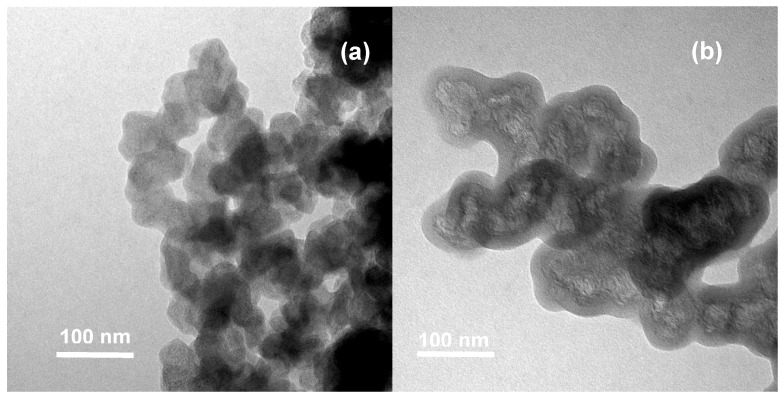
TEM images of (**a**) pristine carbon black and (**b**) HBSPES/CB [9].

We also attempted to develop heterogeneous catalysts by immobilizing HBSPES and LSPES onto carbon black (HBSPES/CB and LSPES/CB). The polymers were dissolved in *N*,*N*-dimethylformamide (DMF) or nitrobenzene and stirred in the presence of carbon black (Ketjen Black EC600JD, Lion Specialty Chemicals, Tokyo, Japan), and the resulting powders were washed with DMF. The ion-exchange capacities (IECs) of the HBSPES and LSPES were 2.1 and 2.5 mmol g^−1^ before the immobilization and decreased to 0.89 and 0.50 mmol g^−1^, respectively. The decrease corresponded to polymer loadings of 39 and 20 wt %, respectively. The difference in polymer loading was most likely because hyperbranched polymers have a denser architecture than their linear analogues [14,15,16]. Figure 2 shows the transmission electron microscopy (TEM) images of pristine carbon black and HBSPES/CB. Compared with that of untreated carbon black, the particle size of HBSPES/CB seemed slightly larger, and HBSPES/CB seemed to have a uniform polymer layer.

### 2.2. Homogenous Catalytic Reactions by HBSPES

**Figure 3:**
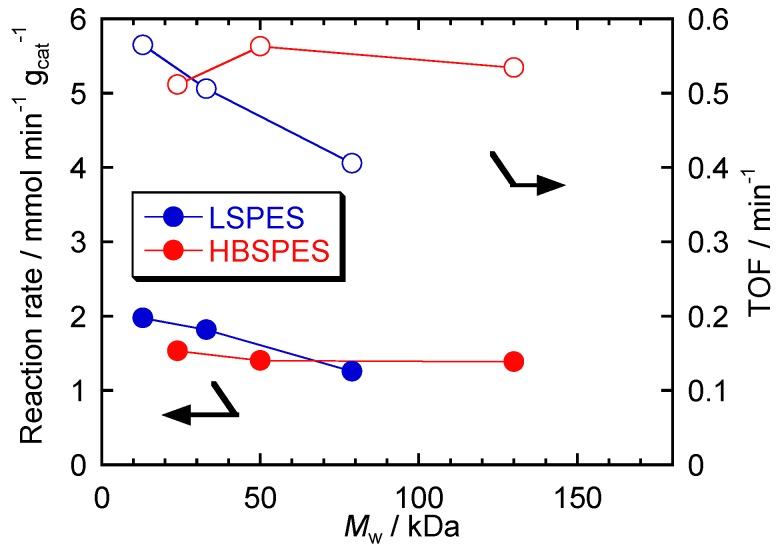
Normalized reaction rates and apparent turnover frequencies of homogeneous catalytic reactions for the esterification of 1-butanol (32 mmol) and acetic acid (1.8 mmol) at 65 °C for 0.5 h with various molecular weights of LSPES and HBSPES [10].

The HBSPES and LSPES were tested as catalysts for the esterification reaction between acetic acid and 1-butanol. Low-molecular-weight model catalysts, *p*-toluenesulfonic acid (PTSA) and 4-(phenoxy)benzenesulfonic acid (PBSA), were also tested, and the results are summarized in Table 1. PTSA and PBSA showed the highest turnover frequencies (TOFs; 3.2 and 4.4 min^−1^, respectively), as they work as homogenous catalysts. The TOF of the HBSPES was fairly high (2.7 min^−1^) whereas that of the LSPES was much lower (0.56 min^−1^). The effects of molecular weight on the catalytic activity of the HBSPES and LSPES for the esterification were further studied, as shown in Figure 3. At a relatively low *M*_w_ of less than 20 kDa, the LSPES seemed to exhibit a slight advantage over the HBSPES in terms of the normalized reaction rate. However, the reaction rate over the LSPES clearly decreased as the molecular weight increased. Furthermore, the LSPES with 79 kDa *M*_w_ was partially insoluble in the reaction solvent. For a relatively low molecular weight, the TOFs of the LSPES and HBSPES were quite similar. This is probably because both the LSPES and HBSPES, having such a low molecular weight, dissolve easily in the reaction solution, making the accessibility of the reactant to the sulfonic acid groups. However, the TOF of the LSPES clearly decreased as the molecular weight increased, whereas that of the HBSPES remained steady. This could be due to the inter- and intra-chain aggregation of the LSPES and suggests that the ability of the sulfonic acid groups in the LSPES to access the reactant worsened at high molecular weights. These experimental results indicate that the HBSPES exhibits a steady catalytic activity over a wider range of molecular weights. This is probably because hyperbranched polymers have a good affinity for the reaction solvent given the low entanglement of the polymer chains, which leaves the terminal functional groups well-exposed to the reactants.

### 2.3. Heterogeneus Catalytic Reactions by HBSPES

**Figure 4:**
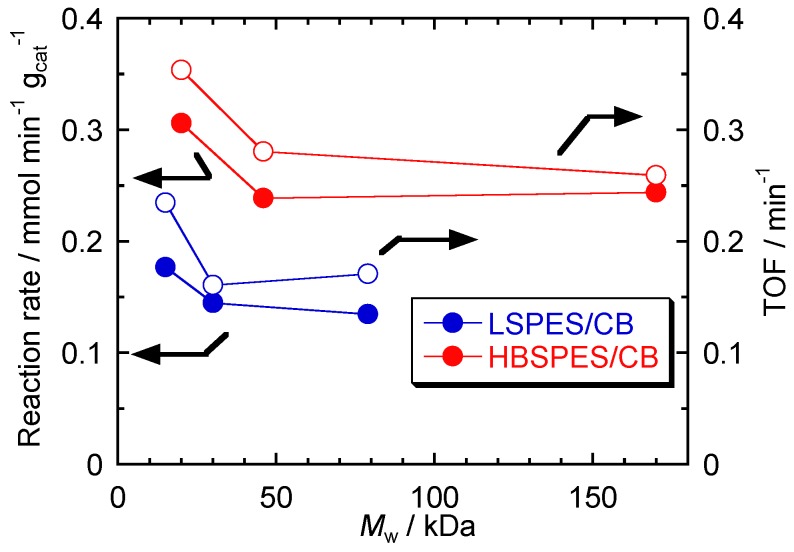
Normalized reaction rates and apparent turnover frequencies of homogeneous catalytic reactions for the esterification of 1-butanol (32 mmol) and acetic acid (1.8 mmol) at 65 °C for 0.5 h [10].

HBSPES/CB and LSPES/CB were tested for a heterogeneous catalytic reaction for the esterification of 1-butanol and acetic acid. The results of these catalysts and the commercial Amberlyst^®^-15 (Sigma-Aldrich, Tokyo, Japan) are compared in Table 1. The TOF of HBSPES/CB was lower than that of the HBSPES, but still better than that of Amberlyst^®^-15 and LSPES/CB. The effects of molecular weight on the catalytic activity of HBSPES/CB and LSPES/CB for the esterification were further studied, as shown in Figure 4. For all of the tested molecular weights, the TOFs of HBSPES/CB were much better than those of LSPES/CB. These results probably reflect the nature of the hyperbranched polymers, which can be immobilized with a higher polymer loading due to their compact architecture, and can afford a higher catalytic activity of the end-groups due to their good affinity for the reaction solution. 

**Figure 5:**
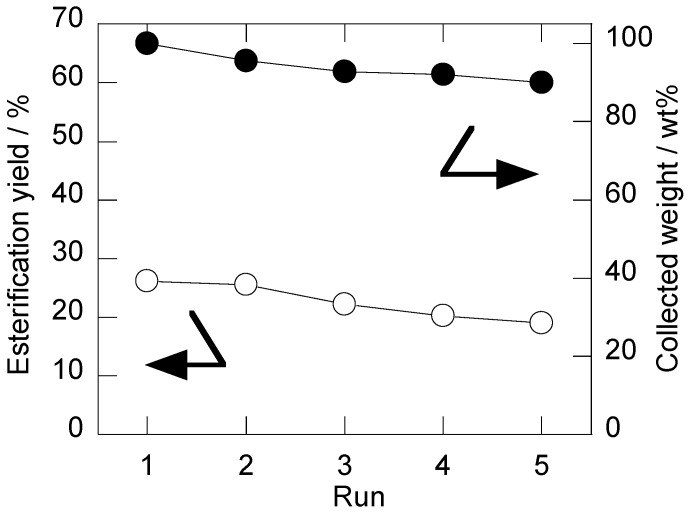
Esterification yields with the fresh catalyst (1st run) and the recycled catalysts (second to fifth run), and the relative amounts of catalyst against the first run. Conditions were identical to those in Table 1, but the catalyst amount was 60 mg and the reaction period was 2.5 h [9].

The recyclability of HBSPES/CB was tested by filtering the reaction mixture and reusing the catalyst powder, and the results are shown in Figure 5 [9]. Although the collection yield of HBSPES/CB was not 100%, over 90% HBSPES/CB was successfully collected, even after five runs. Moreover, the catalytic activity of HBSPES/CB appeared to be fairly stable, so it can be concluded that the stability of HBSPES/CB is quite promising.

### 2.4. Catalytic Reactions Benefitting from High Thermal Stability

**Scheme 2:**
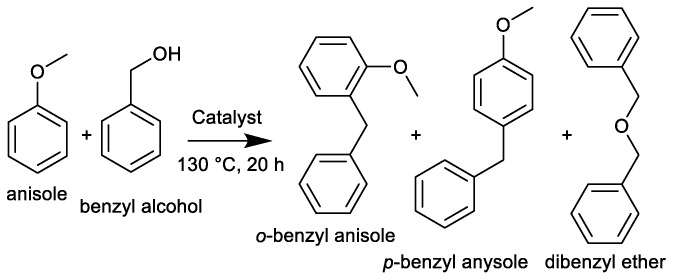
A model reaction at a high temperature, Friedel–Crafts alkylation of anisole.

**Figure 6:**
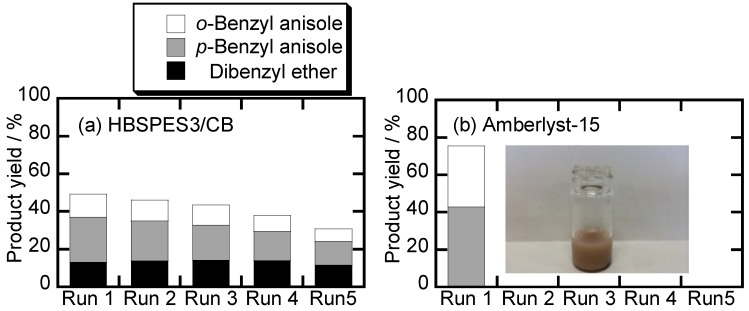
The results of Friedel–Crafts alkylation over (**a**) HBSPES/CB and (**b**) Amberlyst^®^-15. Conditions: anisole 18.5 mmol, benzyl alcohol 1.25 mmol, catalyst 75 mg, 130 °C, 20 h. Inset: the reaction mixture after the first run with Amberlyst^®^-15 [9].

As the aromatic main chains of HBSPESs have a high thermal stability, a higher operating temperature can be expected when compared with that for Amberlyst^®^-15. In this context, the Friedel–Crafts alkylation of anisole with benzyl alcohol was studied using HBSPES/CB, as illustrated in Scheme 2, and the results are summarized in Figure 6. HBSPES/CB showed a reliable catalytic activity for this reaction. Furthermore, the catalyst was successfully collected after the reaction and used subsequently for an additional four runs, although the performance slightly degraded as the run number increased. Amberlyst^®^-15 also exhibited catalytic activity under the same conditions, but the reaction mixture became turbid immediately at the early stage of the reaction, and it was impossible to collect the catalyst. Amberlyst^®^-15 probably decomposed in these reaction conditions and worked as a homogenous catalyst. These experimental results clearly suggest that the stability of the HBSPES/CB catalyst is superior to that of the typical ion-exchange resin under such high temperatures.

### 2.5. Perspective for HBSPES

As mentioned above, HBSPES has been demonstrated as homogenous and heterogeneous catalysts, and the advantage against linear polymers has also been experimentally evidenced. Since sulfonic acid groups can catalyze many catalytic reactions, more various chemical reactions can be performed. The next challenge for HBSPES in catalysts could be the density of active sites, especially in the heterogeneous form. The immunization of HBSPES onto a carbon surface is actually useful, but even higher polymer loadings would be ideal to have a higher ion exchange capacity. The design of the monomers and polymers could also be further improved to increase the number of sulfonic acid groups.

## 3. Hyperbranched Poly(Ether Ketone) for Catalysis

Although HBSPESs work well as acid catalysts for several reactions, the end-groups of HBSPESs are not easy to convert into other functional groups. If aromatic hyperbranched polymers with reactive end-groups that can be modified with various functional units could be developed, the use of hyperbranched aromatic polymers would be further expanded to various catalytic reactions. In this context, we focused on the development of hyperbranched poly(ether ketone) (HBPEK).

### 3.1. Synthesis of HBPEK

Although there are several examples for the synthesis of HBPEK [17,18,19,20,21], those for reactive end-groups such as carboxylic acid are quite limited. Shu and Leu reported the synthesis of HBPEK with carboxylic acid end-groups through the polycondensation of an AB_2_ monomer, 5-phenoxyisophthalic acid [18]. As the two B units were present on the same benzene ring, the activity of the B units was limited by steric hindrance, resulting in a relatively low molecular weight of *M*_w_ of <15 kDa.

**Scheme 3:**
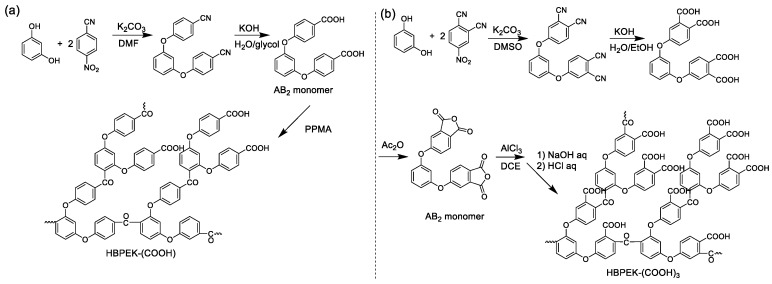
Synthesis routes of hyperbranched poly(ether ketone) with (**a**) carboxylic acid and [22] (**b**) phthalic acid end-groups [23].

In this context, our research group proposed AB_2_ monomers with more reactive B units. Scheme 3a shows the synthesis route of HBPEK with carboxylic acid end-groups [22]. In this AB_2_ monomer, 4,4’-(*m*-phenylenedioxy)-*bis*-(benzenecarboxylic acid), the B units are expected to exhibit a high reactivity due to the high flexibility derived from the ether bonds and less steric hindrance. Indeed, the polycondensation of this AB_2_ monomer in phosphorous pentoxide–methanesulfonic acid (PPMA) resulted in a fairly high *M*_w_ of up to 159 kDa, accompanied by reasonable IECs of 2.2–2.8 mmol g^−1^. The carboxylic acid groups can work as weakly acidic catalysts or can be modified with various functional groups using end-capping reactions to form amide or ester bonds.

Aiming for a higher IEC and more reactive end-groups, another AB_2_ monomer, 1,3-*bis*-(3,4-dicarboxyphenoxy)benzene dianhydride, as shown in Scheme 3b, was also proposed by our research group [23]. This monomer can be polymerized without any byproducts and will leave phthalic acid end-groups, which can afford a high IEC. This polycondensation chemistry has been used in a very limited number of A_2_ + B_3_ samples [24,25], but this was the first example that applied it to the synthesis of HBPEK by the self-polycondensation method employing the Friedel–Crafts reaction for the synthesis of HBPEK. The polycondensation of this monomer in the presence of AlCl_3_ resulted in fairly high *M*_w_ of up to 224 kDa, accompanied by quite high IECs of 6.4–7.4 mmol g^−1^. These phthalic acid end-groups are quite promising as catalytically active sites for the hydrolysis of cellulose [26].

### 3.2. Immobilization of HBPEK onto Supports

**Figure 7:**
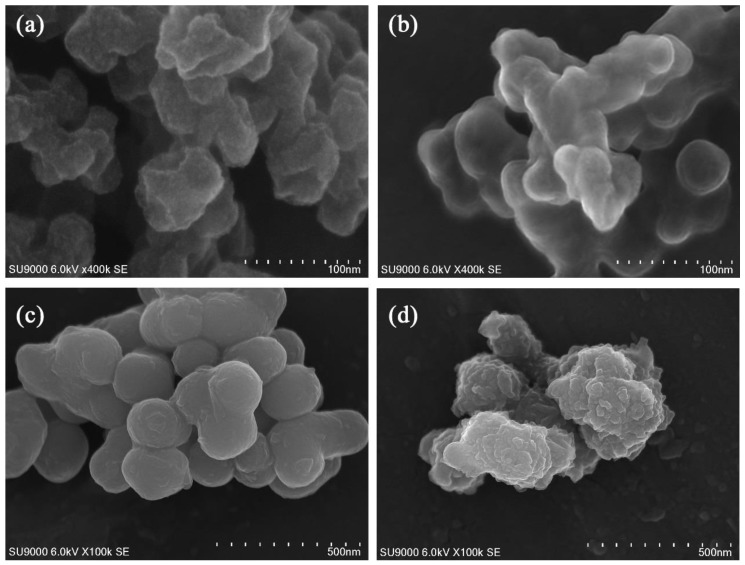
FE-SEM images of (**a**) pristine carbon black; (**b**) HBPEK/CB; (**c**) pristine polyimide nanoparticles; and (**d**) HBPEK/PI [27].

To use HBPEK as a heterogeneous catalyst, the immobilization of HBPEK onto supports such as carbon black [27], polyimide (PI) nanoparticles [27], and graphene [28] has been demonstrated by our research group. Figure 7 shows the field-emission scanning electron microscopy (FE-SEM) images of carbon black and PI nanoparticles before and after the immobilization of HBPEK. We synthesized the PI nanoparticles via precipitation polymerization [29,30,31,32]. HBPEK was treated with these aromatic supports in the presence of PPMA by using the same condensation chemistry as that of the aforementioned polymerization of HBPEK. The carbon black–treated HBPEK (HBPEK/CB) showed a relatively smooth surface, whereas the surface of the pristine CB seemed quite rough due to its microporous structure. The HBPEK treated with PI nanoparticles (HBPEK/PI) also showed a clearly different surface from that of pristine PI. The immobilization of HBPEK was also confirmed from the difference in the IEC and the results of the elemental analysis [27].

### 3.3. Catalytic Reactions with HBPEK

#### 3.3.1. Hydrolysis of Cellulose

The production of renewable chemicals and fuel from biomass has attracted a great deal of attention with regard to a green and sustainable society, and the catalytic conversion of cellulose to glucose is a very important reaction [33,34]. Conventionally, this reaction has been demonstrated using mineral acids [35], but a more environmentally friendly process should be established. Kobayashi et al. demonstrated that several carbon materials with weakly acidic functional groups such as carboxylic acids were promising catalysts for this purpose [26,36]. Such oxygen-containing functional groups are typically introduced by the oxidation of carbon surfaces, but it is generally difficult to precisely control the chemical structures and densities of such functional groups by the surface modification of carbon materials.

**Scheme 4:**
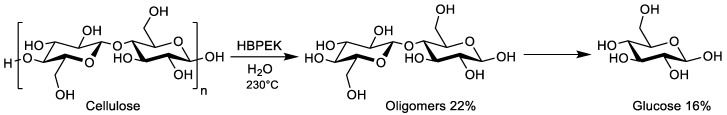
Catalytic conversion of cellulose to glucose.

In this context, as a more well-defined weakly acidic material, the investigation of HBPEK for the catalytic conversion of cellulose to glucose is of interest because HBPEK has numerous carboxylic acid terminals on its aromatic rings. As illustrated in Scheme 4, HBPEK was tested for the hydrolysis of cellulose and certainly exhibited catalytic activity. After treating 324 mg cellulose in the presence of 50 mg HBPEK and 40 mL water, at 230 °C, the conversion of cellulose was 53%, yielding 22% oligomers and 16% glucose [22]. This result suggests that the carboxylic acid groups of HBPEK certainly functioned as weakly acidic sites; however, this catalytic process needs to be further optimized.

#### 3.3.2. Partial Oxidation of Alcohols

The oxidation of primary alcohols to aldehydes is important in terms of the synthesis of chemicals and intermediates. The aerobic oxidation of organic compounds is especially of interest because the reaction consumes oxygen as the oxidizing agent and produces only water as the byproduct. For this purpose, a nitroxyl radical with low toxicity and reversible redox behavior, 2,2,6,6-tetramethylpiperidine-1-oxyl (TEMPO), has been studied [37]. Furthermore, our research group presented a nitric acid–assisted carbon-catalyzed oxidation system (NACOS) using carbon-based materials as metal-free catalysts [38] and demonstrated that TEMPO enhanced this oxidation system [39]. Although TEMPO has typically been demonstrated as a homogenous catalyst, the immobilization of TEMPO onto solid supports such as polystyrene [40], silica [41], and iron oxide [42] has also been reported. These materials are quite promising for establishing heterogeneous reaction systems, which will be more cost-effective and environmentally friendly compared to homogeneous systems.

**Scheme 5:**
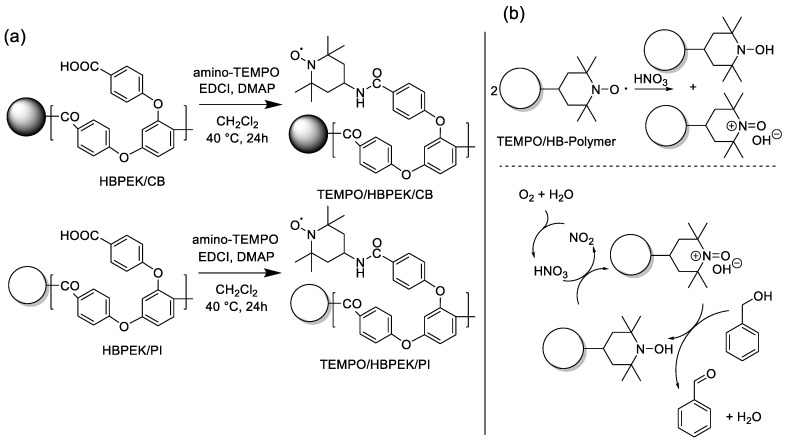
(**a**) Procedure for the modification of HBPEK with TEMPO [27] and (**b**) reaction mechanism of the NACOS, over TEMPO-based catalysts.

In this context, HBPEK could be promising for the immobilization of such catalytically active groups via the modification of the carboxylic acid terminals. Scheme 5a shows the procedures to immobilize the TEMPO units by forming amide bonds. HBPEK was loaded onto carbon black or PI nanoparticles, and the resulting HBPEK/CB and HBPEK/PI were treated with amino-TEMPO, yielding 1.39 and 0.96 mmol g^−1^ of TEMPO loadings, respectively.

These catalysts were tested for the aerobic oxidation of benzyl alcohol in the NACOS, as shown in Scheme 5b, and the results are summarized in Table 2 [27]. The turnover number (TON) of the TEMPO unit was calculated from the yield of benzaldehyde. A control test without the catalyst gave a low yield. Commercial TEMPO, which was a homogeneous system, showed the highest activity, with a TON of 60. The catalytic activity of TEMPO/HBPEK, which was also a homogenous system, was slightly lower, most likely due to the lower mobility of the TEMPO terminal units on HBPEK. Two synthesized heterogeneous catalysts, TEMPO/HBPEK/CB and TEMPO/HBPEK/PI, exhibited a reliable catalytic activity. The performance of TEMPO/HBPEK/CB was slightly better than that of TEMPO/HBPEK/PI, perhaps due to the synergy between TEMPO and CB [39]. Both TEMPO/HBPEK/CB and TEMPO/HBPEK/PI were easy to separate from the reaction solution, and over 90 wt% of the catalysts were successfully collected by filtration.

Another derivative, HBPEK immobilized onto graphene has been demonstrated for a slightly more difficult oxidation reaction, the aerobic oxidation of 2-adamantanol [28]. The oxidation of 2-adamantanol in 1,4-dioxane at 60 °C for 12 h yielded in 18.3% of 2-adamantanone, which corresponded to a TON of 5, accompanied by a good recyclability of the catalyst.

#### 3.3.3. Perspective for HBPEK

Although quite limited kinds of catalytic reactions have been demonstrated, HBPEK can be applied to a wider range of catalytic reactions by introducing various catalytic centers. In particular, the phthalic acid unit is quite reactive and suitable for the post-functionalization. Further studies will be done to expand the applicability of HBPEK.

## 4. Hyperbranched Polyimide (HBPI) for Catalysis

Among the high-performance aromatic polymers, PI can be regarded as the most thermally stable polymer. Therefore, the use of HBPI in catalysis is worth exploring. HBPI can be synthesized by the self-condensation of an AB_2_ monomer [43,44,45] or copolymerization of an A_2_ monomer and a B_3_ monomer [46,47,48]. Such A_2_ + B_3_ polymerizations typically result in cross-linked network polymers [49,50]; however, careful reaction control (monomer ratio, monomer feeding speed, etc.) yields hyperbranched polymers [46], and the remaining terminals can be modified using an end-capping reaction [48].

**Scheme 6:**
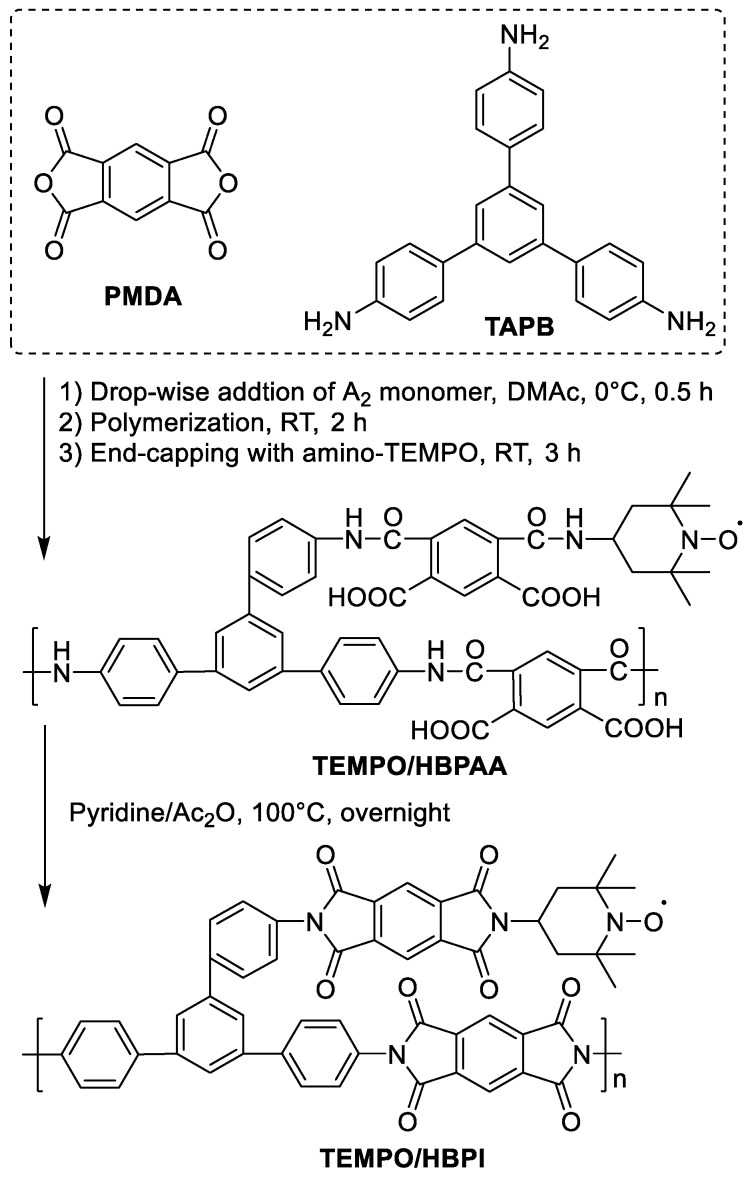
Synthesis of hyperbranched polyimide modified with TEMPO [43,48].

In this study, an A_2_ + B_3_ polymerization of pyromellitic dianhydride (PMDA) and 1,3,5-tris(4-aminophenyl)benzene (TAPB) was selected, and the phthalic acid unit was modified with amino-TEMPO to develop a catalytically active PI (TEMPO/HBPI) [48], as shown in Scheme 6. This synthesis procedure yielded HBPI modified with 1.2 mmol g^−1^ of TEMPO units. Table 2 shows the results of the aerobic oxidation of benzyl alcohol using this TEMPO/HBPI, in comparison with the catalytic activity of HBPEK-based catalysts. TEMPO/HBPI exhibited a reliable catalytic activity, with a TON of 51, which was higher than those with HBPEK-based catalysts. Interestingly, this HBPI could be easily collected by filtration; therefore, it could be used as a heterogeneous catalyst without immobilizing the polymer onto carbon black. This is probably because this hyperbranched polymer is partially cross-linked since it was obtained from an A_2_ + B_3_ polymerization. Since the phthalic acid end-groups have a high reactivity, various other catalytically active sites could be investigated in the near future.

Compared to HBSPES and HBPEK, the affinity of the polyimide backbone chains to typical solvent systems tends to be poor, which will be the main problem for HBPI in catalysis. However, if the high thermal stability of polyimide could be wisely utilized, more challenging catalytic reactions might be possible, benefitting from higher reaction temperatures.

## 5. Conclusions and Future Prospects

Various hyperbranched aromatic polymers, HBSPES, HBPEK, and HBPI, have been designed for use in catalysis. One clear advantage of the application of these hyperbranched polymers is the high reactivity of the end-groups, which are well-exposed due to the low entanglement of the main chains. These polymers can be used as heterogeneous catalysts; moreover, a good catalytic activity comparable to that of homogeneous catalysts can be expected since hyperbranched polymers have a good affinity for many solvents. In other words, hyperbranched polymers can combine the advantages of homogenous and heterogeneous catalysts: high catalytic activity and ease of separation. We demonstrated several model catalytic reactions in this article, but other challenging catalytic reactions will be investigated by introducing various catalytically active sites. In addition, electro-catalytic processes using hyperbranched polymers could be our next area of interest, as the syntheses of several π-conjugated hyperbranched polymers have been recently demonstrated [51,52,53,54]. Further studies would be able to develop more attractive and exciting catalytic systems, which we believe will contribute to a sustainable future by green chemistry.

## Figures and Tables

**Table 1:** Results of catalytic esterification reaction [9].

Catalyst	IEC mmol g^−1^	*w*^1^ mg	*t*^2^ h	Yield ^3^ %	TOF ^4^ min^−1^
Blank	-	-	2.5	4	-
Ketjen black	-	20	2.5	4	-
PTSA	5.3	10	0.5	26	3.2
PBSA	4.0	10	0.5	21	3.4
HBSPES ^5^	2.1	20	0.5	18	2.7
HBSPES/CB	0.89	20	2.5	13	0.67
LSPES ^6^	2.5	20	2.5	25	0.56
LSPES/CB	0.50	20	2.5	5	0.13
Amberlyst^®^-15	4.7	20	2.5	21	0.24

^1^ Amount of the catalyst. ^2^ Reaction period. ^3^ Esterification of 1-butanol (0.02 mol) and acetic acid (0.02 mol) at 65 °C. ^4^ Calculated from the IEC and the esterification yield. ^5^ *M*_w_: 91 kDa. ^6^ *M*_w_: 18 kDa.

**Table 2:** Results of aerobic oxidation of benzyl alcohol to benzaldehyde ^1^ [27,43].

Catalyst	TEMPO Loading mmol g^−1^	*w*^2^ mg	Yield %	Selectivity %	TON
Blank	-	-	1	50	-
TEMPO	6.4	12.5	48	98	60
TEMPO/HBPEK	2.0	40	37	98	46
TEMPO/HBPEK/CB	1.3	62	23	86	27
TEMPO/HBPEK/PI	1.0	80	18	>99	22
TEMPO/HBPI	1.2	20	11	>99	51

^1^ Benzyl alcohol (10 mmol) was treated in the presence of nitric acid (0.2 mmol) and the TEMPO-based catalyst at 90 °C for 2 h with an oxygen balloon. ^2^ The amount of the catalyst.
